# Mammalian CBX7 isoforms p36 and p22 exhibit differential responses to serum, varying functions for proliferation, and distinct subcellular localization

**DOI:** 10.1038/s41598-020-64908-2

**Published:** 2020-05-15

**Authors:** Kyu-Won Cho, Mark Andrade, Yu Zhang, Young-sup Yoon

**Affiliations:** 1 Department of Medicine, Division of Cardiology, Emory University School of Medicine, Atlanta, GA 30322 USA; 2 Severance Biomedical Science Institute, Yonsei University College of Medicine, Seoul, South Korea; 3 Lead Contact Department of Medicine, Division of Cardiology, Emory University School of Medicine, Atlanta, GA 30322 USA

**Keywords:** Cell division, Gene expression

## Abstract

CBX7 is a polycomb group protein, and despite being implicated in many diseases, its role in cell proliferation has been controversial: some groups described its pro-proliferative properties, but others illustrated its inhibitory effects on cell growth. To date, the reason for the divergent observations remains unknown. While several isoforms for CBX7 were reported, no studies investigated whether the divergent roles of CBX7 could be due to distinct functions of CBX7 isoforms. In this study, we newly identified mouse CBX7 transcript variant 1 (mCbx7v1), which is homologous to the human CBX7 gene (hCBX7v1) and is expressed in various mouse organs. We revealed that mCbx7v1 and hCBX7v1 encode a 36 kDa protein (p36^CBX7^) whereas mCbx7 and hCBX7v3 encode a 22 kDa protein (p22^CBX7^). This study further demonstrated that p36^CBX7^ was localized to the nucleus and endogenously expressed in proliferating cells whereas p22^CBX7^ was localized to the cytoplasm, induced by serum starvation in both human and mouse cells, and inhibited cell proliferation. Together, these data indicate that CBX7 isoforms are localized in different locations in a cell and play differing roles in cell proliferation. This varying function of CBX7 isoforms may help us understand the distinct function of CBX7 in various studies.

## Introduction

Cell proliferation is a finely tuned process and its dysregulation is associated with many diseases including cancers, cardiovascular diseases, neurodegenerative diseases, immune diseases, and congenital disorders^1,2^. While genes directly control cell growth and division as evidenced by genetic alterations in proto-oncogenes or tumor suppressor genes in various cancers^3^, epigenetics control cell proliferation by modulating accessibility of transcription machinery to DNA^4^ and the resulting gene expression patterns^5^. Thus, genome-wide epigenetic modifications are one of the major hallmarks of cells with dysregulated proliferation^6,7^ and epigenetic changes are causative factors for various diseases where cell proliferation is aberrant^8^.

One class of epigenetic regulators is polycomb group (PcG) proteins. First discovered in fruit flies, they are known to govern cell proliferation^9,10^. They epigenetically control transcription of cell cycle-regulatory genes such as CDKN2A^11^, PTEN^12^, and c-MYC^13^. The canonical mechanism of PcG-mediated epigenetic silencing involves coordinated actions of two major types of polycomb repressive complex (PRC), PRC1 and PRC2^14^. PRC2 initiates the repressing process by tri-methylation of Histone 3 tail (H3K27me3)^15^. PRC1 is then recruited and stabilizes this silencing process via mono-ubiquitination of H2A tail (H2Aub)^16^. Finally, H2Aub serves as a binding site for PRC2 which further propagates the H3K27me3 repressive histone mark on H2Aub nucleosomes, generating a positive feedback loop^17^.

CBX family proteins are subunits of PRC1 and recognize trimethylated histone tail, thereby determining target specificity^18,19^. There are five mammalian orthologues in the CBX family: CBX2, 4, 6, 7 and 8. CBX family members are characterized by a chromodomain that binds to trimethylated histone tail (H3K27me3)^18^. They also have a polycomb repressor (Pc) box that interacts with other polycomb proteins^20^.

Among CBX family members, CBX7 has been suggested to regulate cell proliferation;^21^ however, there have been conflicting reports on its role on cellular proliferation. CBX7 was first known for its function in extending cellular life span and its expression was detected in brain, lung, liver, skeletal muscle, kidney, and heart^21^. Afterwards, CBX7 was reported to function as an oncogene since it was highly upregulated in prostate cancer, lymphoma, and gastric cancer^22–24^. In these cancers, CBX7 repressed senescence- and apoptosis-related genes such as Ink4a/Arf and Trail. On the other hand, other investigators claimed that CBX7 serves as a tumor suppressor gene in certain types of cancers including breast cancer, pancreatic cancer, lung cancer, thyroid cancer, colon cancer, bladder cancer and brain cancer^21–23,25–29^. They showed that CBX7 represses transformation- and cell cycle-related genes such as Wnt/β-catenin and Cyclin E1, respectively. To reconcile these divergent observations, it was suggested that CBX7 may function in tissue- and context-specific manners^30^. However, the mechanism underlying this discrepancy on cell proliferation is unknown.

Interestingly, while several isoforms for CBX7 were reported, no studies investigated whether the divergent roles of CBX7 in different cancers could be caused by different CBX7 isoforms. To date, three transcript variants for human CBX7 gene and one sequence for mouse Cbx7 gene were identified and available at the National Center for Biotechnology Information (NCBI). Automated computational analysis of genomic sequences predicted 8 sequences for mouse Cbx7 variants, but none of them were verified. A previous report showed that human and mouse CBX7 proteins have similar functions such as normal cell growth, while having different molecular weights^21^. However, no studies compared expression patterns and the role of CBX7 isoforms in cell proliferation between human and mouse.

Here we report that CBX7 isoforms display distinct characteristics in their molecular weight, their intracellular location, and their effects on cellular proliferation. We newly identified mouse CBX7 variant 1 (mCbx7v1) and demonstrated that it is homologous to human CBX7 variant 1 (hCBX7v1). The original mouse CBX7 (mCbx7) was found to be homologous to human CBX7 variant 3 (hCBX7v3). hCBX7v1 and mCbx7v1 produced a 36 kDa protein (p36^CBX7^) whereas hCBX7v3 and mCbx7 encoded a 22 kDa protein (p22^CBX7^). p36^CBX7^ was endogenously expressed in proliferating cells whereas p22^CBX7^ was induced by serum starvation. Canonical 36^CBX7^ was localized to the nucleus but its overexpression did not affect cell proliferation. However, p22^CBX7^ was largely localized to the cytoplasm and its overexpression inhibited cell proliferation. These distinct characteristics of CBX7 isoforms may facilitate our understanding of the roles of CBX7 in cell proliferation in different contexts.

## Results

### Mouse Cbx7 transcript variant 1 is homologous to human CBX7 transcript variant 1

**Figure 1:**
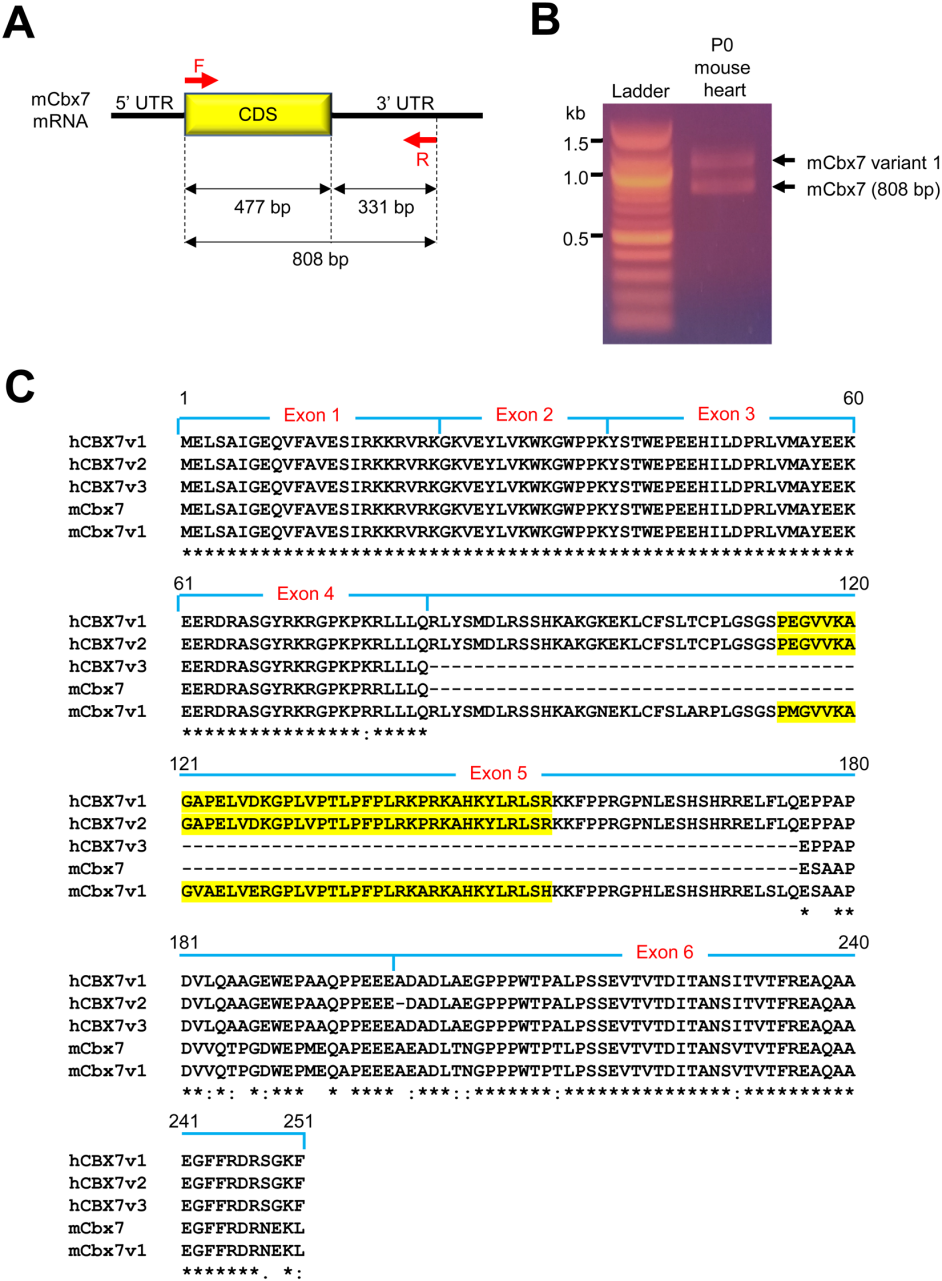
Molecular cloning of mCBX7v1. (**A**) A schematic showing primer binding regions in mouse CBX7 mRNA. The forward primer was designed to bind 5′ region of CDS whereas the reverse primer was designed to binds the 3′UTR. The expected size of the amplified DNA is 808 bp. (**B**) Electrophoresis of the PCR product in an agarose gel. PCR was performed with cDNA from the neonatal (P0) mouse heart. (**C**) Protein sequence alignment of human and mouse CBX7 isoforms. Multiple sequence alignment was performed using CLUSTAL 2.0.12. MDR (medium chain reductase) superfamily domain is in yellow.

To determine the role of CBX7 in proliferation of cardiovascular cells, we constructed plasmids containing CBX7 cDNAs. For molecular cloning of mouse CBX7 cDNAs, we designed primers to amplify the full length CBX7 cDNA of 808 bp, including 477 bp of coding sequence (CDS) and 331 bp of the 3′ untranslated region (3′ UTR) (Fig. 1A). The forward primer binds to the 5′ region of protein coding sequence (CDS) whereas the reverse primer binds to the 3′UTR. Therefore, the amplified DNA should be 808 bp. However, PCR with cDNA from the neonatal mouse heart showed an additional band right above the expected band at ~1.1 kb. We speculated that this band could be a transcript variant of the CBX7 gene (Fig. 1B). To confirm that the upper band is a CBX7 variant gene, we performed gene cloning and found that it was indeed a transcript variant of the CBX7 gene. Since our cloned sequence was homologous to the “human CBX7 transcript variant 1” at the National Center for Biotechnology Information (NCBI), we named this gene mouse Cbx7 transcript variant 1 (mCbx7v1) (GenBank Accession #: MN581682). Three isoforms for human CBX7 gene have been reported. To compare the differences between human and mouse isoforms, we aligned protein sequences for human CBX7 isoforms (hCBX7v1–3) and mouse Cbx7 variants (mCbx7 and mCbx7v1) (Fig. 1C). hCBX7v1 and hCBX7v2 showed 99.6% identity. The only difference was that hCBX7v2 lacks an alanine at the 200th residue, which exists in all other variants. hCBX7v1 and mCBX7v1 were composed of 251 amino acids (a.a.) whereas hCBX7v3 and mCBX7 were composed of 158 a.a. Both human and mouse CBX7 genes have six exons. In all human and mouse CBX7 variants, all exons except for exon 5 showed identical or similar sequences: 100% identity in exons 1–3, 95.5% in exon 4 (human vs. mouse), and 86.3% identity in exon 6 (human vs. mouse, except for hCBX7v2). hCBX7v1, hCBX7v2 and mCbx7v1 have a longer exon 5 with 93 extra amino acids, compared to hCBX7v3 and mCbx7. These results indicate that mouse Cbx7 variant (mCbx7v1) is homologous to human CBX7 (hCBX7v1). We next investigated the role of these additional sequences in exon 5. We ran the domain search program at the NCBI website (https://www.ncbi.nlm.nih.gov/Structure/cdd/wrpsb.cgi). The results showed that exon 5 of CBX7 contains an MDR (medium chain reductase) superfamily domain (yellow color in Fig. 1C). The MDR group exhibits dehydrogenase and reductase activities for numerous substrates^31^. However, the role of the MDR group in the nucleus has not been reported.

We summarized the comparison of CBX7 isoform sequences available at NCBI and Ensembl in Figure S1A. We found that mouse CBX7 transcript variant 1 was highly similar to 201, but not identical (98.4% of identity) (Figure S1B). Other CBX7 isoforms were the same between NCBI and Ensembl. There were two different transcripts for mouse CBX7 with 158 a.a. (202 and 203) at Ensembl although the protein sequence is identical. We added alignment of protein sequences gained from Ensembl together with the sequence we identified (Figure S1C).

To examine whether the long (251 a.a.) and short isoform (158 a.a.) of CBX7 are present in other species, we searched the gene at the NCBI database. We found that both long and short isoforms are present in most mammals but not in other species such as birds, amphibians, and bony fish. We aligned protein sequences of several mammals and found that each isoform is highly homologous among mammals including human, monkey, mouse, rat, dog, cattle, sheep, pig, and horse (Figure S2A,B).

To predict nuclear localization signals (NLSs), we used cNLS Mapper, a prediction program^32^ (Figure S3). The program predicted 2-3 NLSs in between the 63^rd^ and 145^th^ amino acids in Exons 4 and 5 of the long isoforms (hCBX7v1 and mCBX7v1). On the other hand, the short isoform did not have any NLS. These results suggest that the long isoform is localized in the nucleus whereas the short isoform, lacking NLS, remains in the cytoplasm.

### Two mCbx7 transcript variants are expressed in various organs

**Figure 2:**
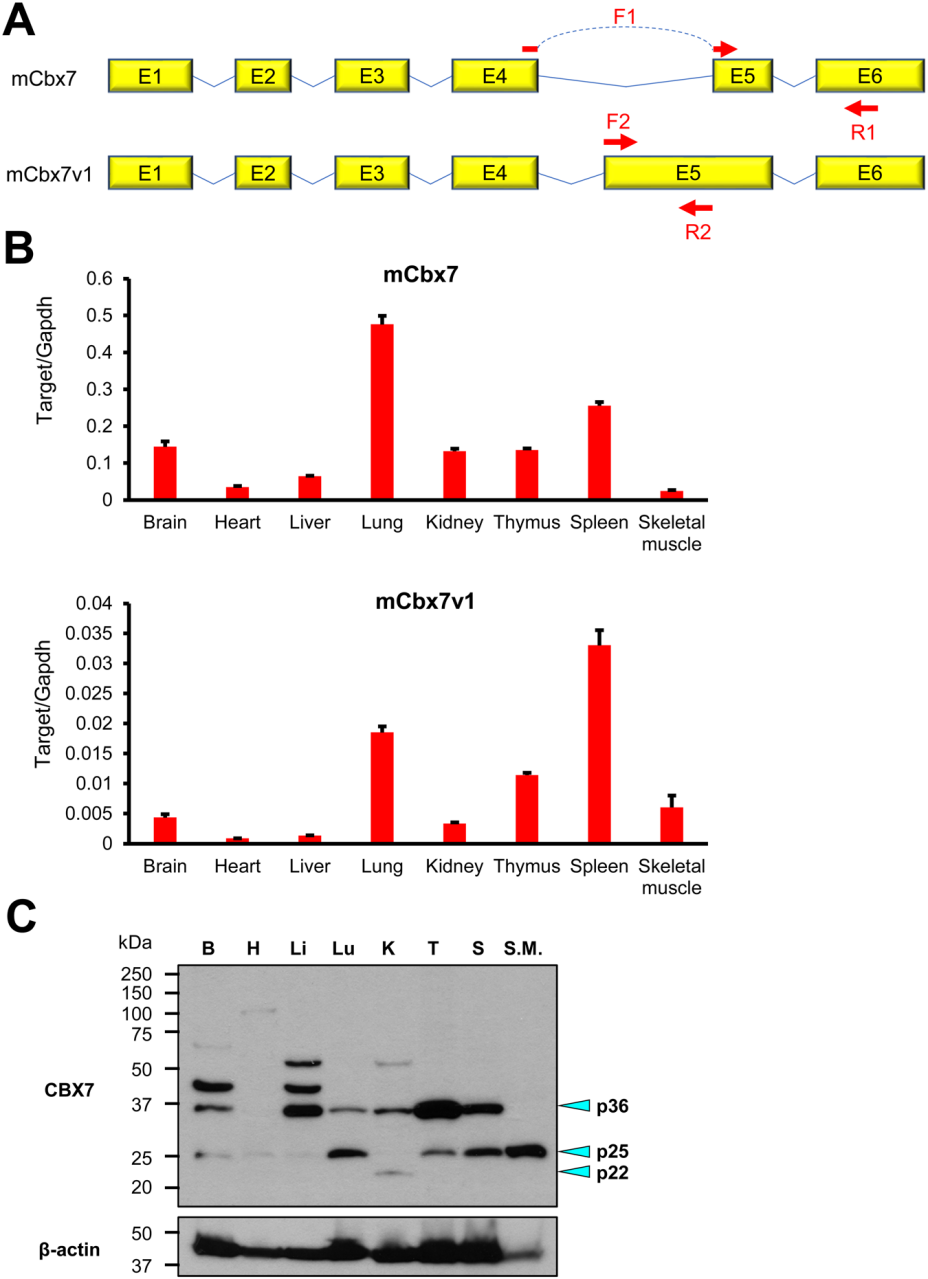
Expression of mCbx7 and mCbx7v1 in various tissues. (**A**) A schematic showing primer binding regions in mCbx7 and mCbx7v1 mRNAs. To detect mCbx7 mRNA, the forward primer (F1) was designed to bind both the 3′ region of exon 4 (E4) and the 5′ region of E5 (upper) and the reverse primer (R1) was designed to bind E6. To detect mCbx7v1 mRNA, the forward and reverse primers (F2 and R2) were designed to bind E5 region that exist only in mCbx7v1 gene but not mCbx7 (lower). (**B**) Representative qRT-PCR results with cDNAs from adult (3-month-old) mouse tissues such as brain, heart, liver, lung, kidney, thymus, spleen and skeletal muscle. N = 3. (**C**) Representative western blot results with whole lysates from adult (3-month-old) mouse tissues. B, brain; H, heart; Li, liver; Lu, lung; K, kidney; T, thymus; S, spleen; S.M., skeletal muscle. Indicated bands represent proteins encoded by mCbx7v1 (p36) and mCbx7 (p25 and p22) based on predicted molecular weights.

Previous reports showed that various organs expressed CBX7^21^. To examine the expression profile of the CBX7 variants in different organs, we designed primers that can differentially detect mCbx7 and mCbx7v1 (Fig. 2A). For detection of mCbx7, the forward primer F1 was designed in the border of exon 4 and 5 and the reverse primer R1 was designed in exon 6. For detection of mCbx7v1, the forward (F2) and reverse (R2) primers were designed to amplify the additional sequence in exon 5 that exists only in mCbx7v1. Through qRT-PCR, we found that both mCbx7 and mCbx7v1 were detected in all organs that we checked: brain, heart, liver, lung, kidney, thymus, spleen, and skeletal muscle (Fig. 2B). In all organs, expression of mCbx7 was higher (4 to 47 times) than mCbx7v1, but the expression patterns were similar.

To examine protein levels of CBX7 isoforms, we extracted the protein from different mouse tissues and performed western blot with an anti-CBX7 antibody. The results showed different expression patterns of the long and short CBX7 isoforms depending on the tissues (Fig. 2C). The long isoform was observed at 36 kDa (p36) whereas the short isoform was seen at 25 kDa (p25) and 22 kDa (p22). p36 was predominant in the brain, liver, kidney, and thymus whereas p25 was predominant in the heart, lung, and skeletal muscle. p36 and p25 were expressed at a similar level in the spleen. The kidney showed p22 instead of p25. qRT-PCR data indicated that the short isoform (p25/p22) was predominant in most tissues. However, western blot data showed that p36 was predominant or similar to p25/p22 in multiple tissues. Predominant protein expression of p36 with lesser mRNA levels could be due to increased protein stability and half-life of p36 via post-translational modifications such as sumoylation^33^ and phosphorylation^34^. Furthermore, protein tertiary or quaternary structures can affect protein half-life^35^. For instance, ubiquitination sites could be preferentially masked in the tertiary or quaternary structure of p36 protein preventing it from degradation through the ubiquitin proteasome pathway (UPP).

### hCBX7v1 and mCbx7v1 encode a 36 kDa protein whereas hCBX7v3 and mCbx7 encode a 22 kDa protein

**Figure 3:**
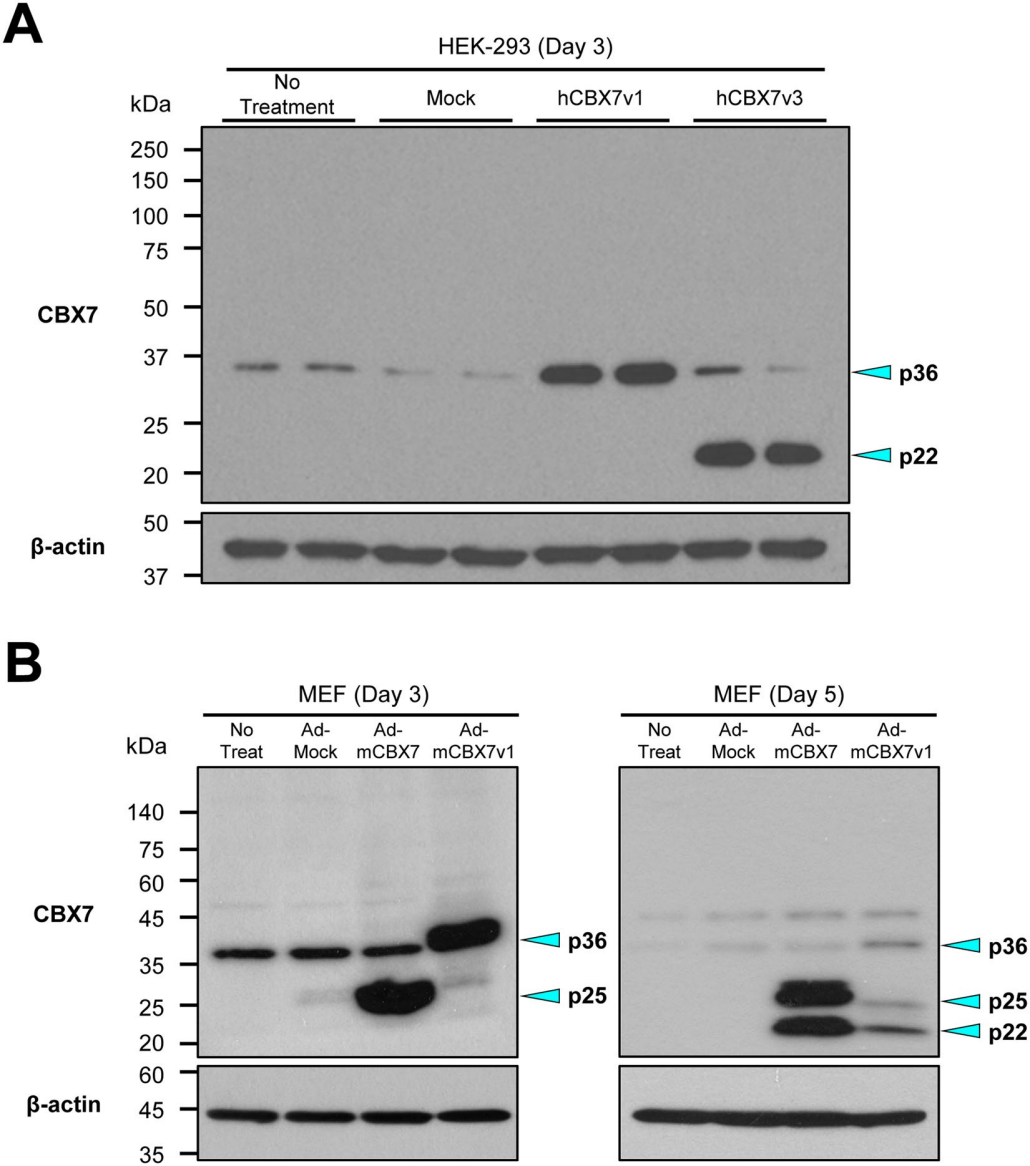
Overexpression of CBX7 isoforms in human and mouse cells. (**A**) A representative western blot image showing overexpression of human CBX7 isoforms in a human embryonic kidney cell line (HEK-293). Plasmids inducing overexpression of hCBX7v1, hCBX7v3, and an empty plasmid construct (Mock) were delivered into HEK-293 cells via liposome-mediated transfection. Non-treated cells and mock-treated cells were used as negative controls. Whole cell lysates were subjected to western blot using an anti-CBX7 antibody. Anti-β-actin antibody was used for internal loading control. (**B**) A representative western blot image showing overexpression of mouse CBX7 isoforms in mouse embryonic fibroblasts (MEFs). MEFs were infected with adenoviral particles inducing overexpression of mCBX7 (Ad-mCBX7), mCBX7v1 (Ad-mCBX7v1) and an empty viral construct (Ad-Mock) for 3 days (left) and 5 days (right). Non-infected cells and Ad-Mock-infected cells were used as negative controls. Whole cell lysates were subjected to western blotting using an anti-CBX7 antibody. Anti-β-actin antibody was used for internal loading control.

To characterize CBX7 isoforms, we overexpressed them in human and mouse cells (Fig. 3 and Supplementary Figure 1). As a human cell study, we transfected human embryonic kidney cells (HEK-293) with each plasmid vector containing hCBX7v1 and hCBX7v3 constructs (Fig. 3A) and performed western blots. Both non-treated- and mock-transfected samples showed a single 36 kDa band with weak intensity. The plasmid containing the hCBX7v1 construct generated a single 36 kDa band with strong intensity. The plasmid inducing overexpression of hCBX7v3 produced two bands, a 22 kDa band with strong intensity and a 36 kDa band with weak intensity.

For a mouse cell study, we generated two kinds of adenoviral particles that induce overexpression of mCbx7 (Ad-mCbx7) and mCbx7v1 (Ad-mCBX7v1), respectively. We infected MEFs with these viral particles and performed western blots 3 and 5 days later (Fig. 3B). At Day 3, both non-treated cells and cells infected with Ad-Mock (the adenoviral particle containing no protein coding sequence) showed a single 36 kDa band with intermediate intensity. Ad-mCBX7 produced a 25 kDa band with strong intensity in addition to the 36 kDa band. Ad-mCBX7v1 generated a single 36 kDa band with strong intensity. At Day 5, Ad-mCbx7 generated a 22 kDa band with strong intensity in addition to the 25 kDa band, suggesting the involvement of post-translational modifications of mCBX7 such as phosphorylation^36^. This reduction of molecular weight from 25 kDa to 22 kDa implies that mCBX7, which was phosphorylated (25 kDa) at Day 3, underwent dephosphorylation, producing the native form (22 kDa) at Day 5. On the other hand, the 36 kDa band generated by Ad-mCbx7v1 became weaker at Day 5, suggesting a short half-life of the 36 kDa protein or negative feedback mechanisms. Furthermore, Ad-mCbx7v1 generated the 25 kDa and 22 kDa bands with weak intensity at Day 5. This suggests that the excessive amount of the 36 kDa protein (at Day 3) triggers a certain pathway which induces expression of the 25 kDa and 22 kDa proteins. The predicted molecular weights (based on amino acid sequence) of mCBX7v1 and mCBX7 is 28 kDa and 18 kDa, respectively. Together, these results indicate that the 36 kDa protein is encoded by hCbx7v1 and mCBX7v1 and is endogenously expressed in proliferative human and mouse cells. We referred to this protein as p36^CBX7^. On the other hand, the 22 kDa protein is encoded by hCBX7v3 and mCbx7 and is not expressed in normal cell culture conditions. We defined this protein as p22^CBX7^. We measured expression levels of the isoforms by densitometric analysis with the western blot data in Fig. 3 and the protein levels of ectopically introduced CBX7 isoforms were similar to each other.

### Proliferative cells express p36CBX7 whereas serum-starved cells express p22CBX7 in both human and mouse

**Figure 4:**
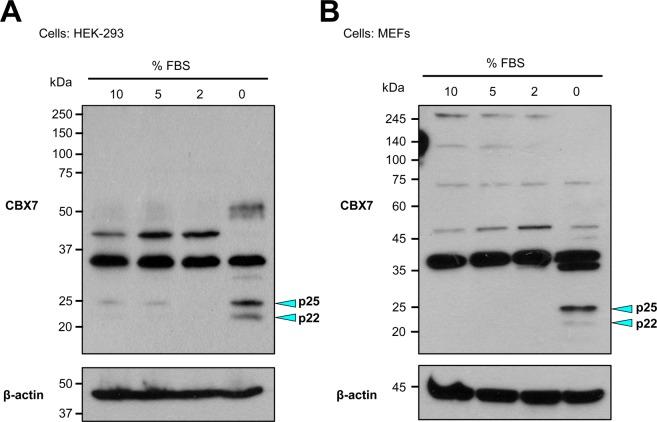
Induction of p22^CBX7^ isoforms in serum-starved mouse and human cells. (**A**) A representative western blot with whole cell lysates of HEK-293 cultured in different serum concentrations (10%, 5%, 2%, and 0%) for 48 hrs. (**B**) A representative western blot with whole cell lysates of MEFs cultured in different serum concentration (10%, 5%, 2%, and 0%) for 48 hrs.

We then wondered what conditions cause p22^CBX7^ to be expressed. Since both HEK-293 cells and MEFs are proliferative and do not express p22^CBX7^, we suspected that non-proliferative cells may express p22^CBX7^. To test this idea, we cultured HEK-293 cells and MEFs with different serum concentrations, including 10, 5, 2, and 0 percent for 2 days and conducted western blots for p36^CBX7^and p22^CBX7^. We found that p36^CBX7^ was expressed in all conditions, but p22^CBX7^ was expressed under serum starvation (0% of FBS) in both HEK-293 cells and MEFs (Fig. 4A-B and Supplementary Figure 2). These results imply that the role of p22^CBX7^ is associated with repressed cell proliferation or cellular quiescence.

MEFs cultured without FBS showed an unexpected band at 35 kDa (Fig. 4B). The origin of this band is unknown, and we speculate that there are three possibilities. First, this band might appear as a result of post-translational modification of p36^CBX7^. A previous report showed that CBX7 is phosphorylated^36^. Thus, this band at 35 kDa could be a dephosphorylated form of p36^CBX7^. Second, it could be a cleaved form of CBX7 protein in response to serum starvation. Reports showed that serum deprivation induces cleavage of proteins including PARP^37^, Emerin^38^, and eIF4G^39^. Finally, it could be another CBX7 isoform generated by alternative splicing.

### p22CBX7 but not p36CBX7 inhibits cell proliferation in both human and mouse cells

**Figure 5:**
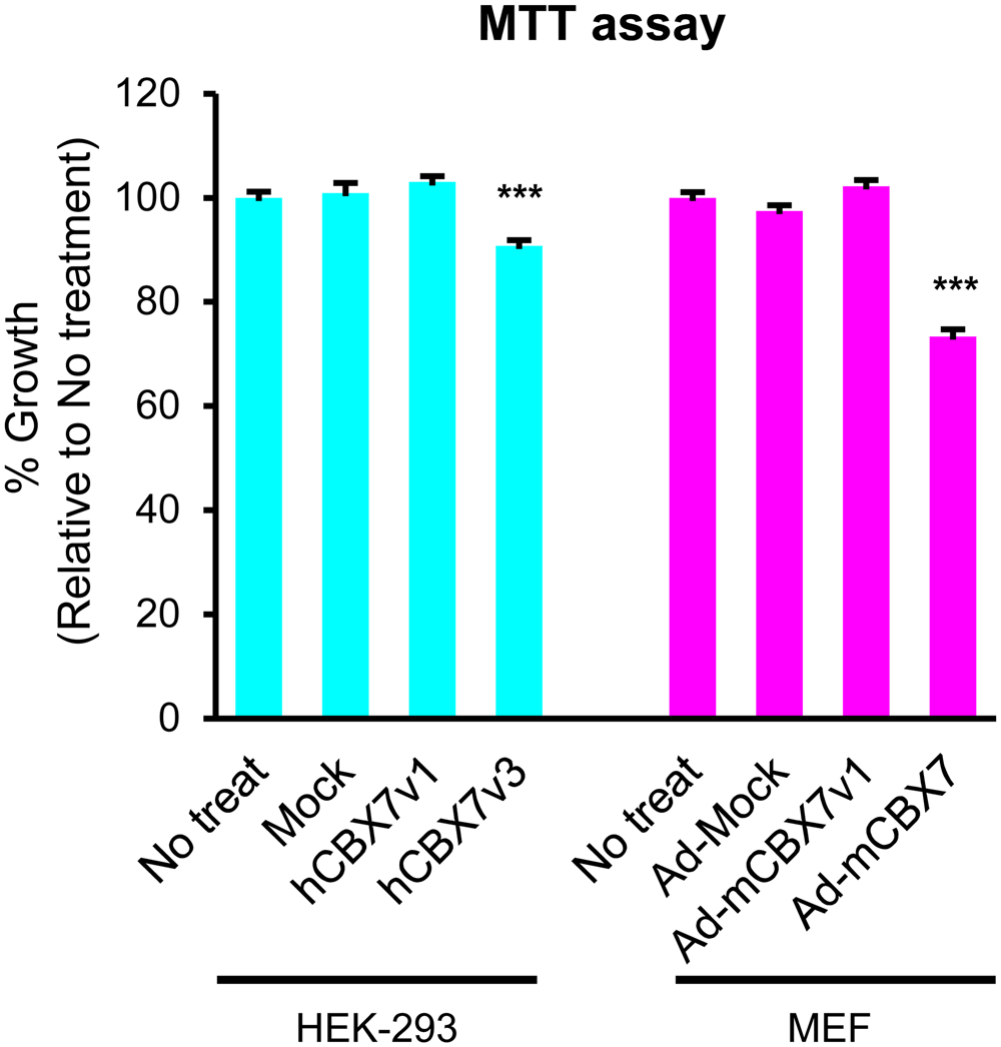
Repression of cell proliferation by p22^CBX7^ isoforms in human and mouse cells. Representative MTT assay results. CBX7 isoforms were overexpressed in HEK-293 cells and MEFs via either liposome-based transfection or adenoviral transduction, respectively. Cells were cultured for 72 hrs and subjected to an MTT assay. Error bars: standard error of mean. One-way ANOVA was performed followed by a Tukey HSD test. N = 15–20 (each group), ***P < 0.001. Each experiment was repeated three times.

To determine the roles of the two CBX7 isoforms in cell proliferation, we overexpressed them in HEK-293 cells and MEFs and performed MTT assay. Overexpression of hCBX7v1 did not affect cell proliferation (Fig. 5, left). However, overexpression of hCBX7v3 reduced proliferation of HEK-293 cells by 10% compared to the controls (no-treatment or mock-plasmid transfected cells). Overexpression of mCBX7v1 did not affect cell proliferation in MEFs. However, overexpression of mCBX7 reduced proliferation of MEFs by 26% compared to the non-infected condition (Fig. 5, right). These results suggest that p22^CBX7^ but not p36^CBX7^ inhibits cell proliferation.

### 36CBX7 is localized to the nucleus whereas p22CBX7 is localized to the cytoplasm in human and mouse cells

**Figure 6:**
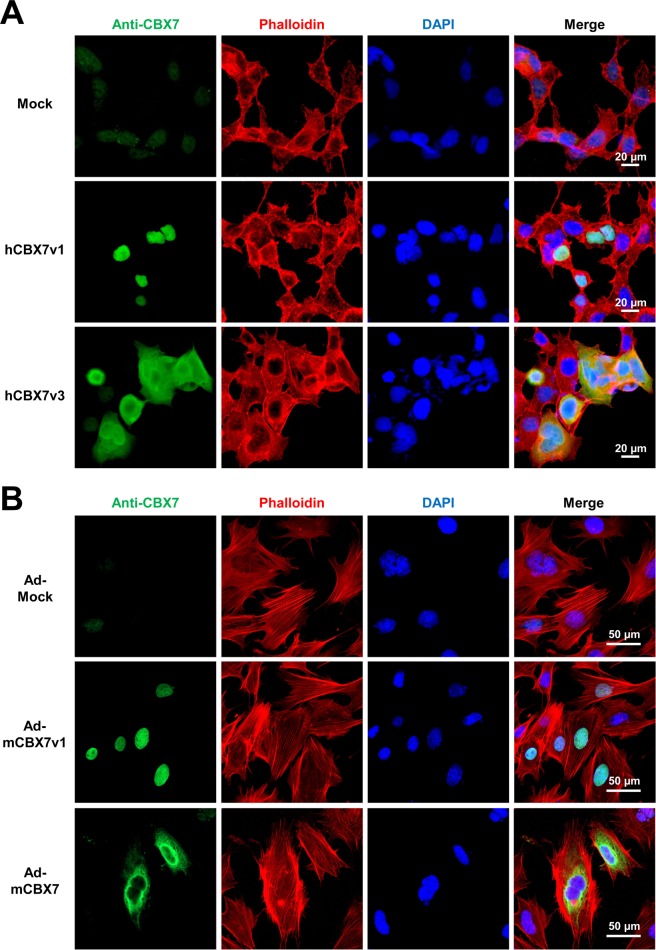
Distinct subcellular localization of CBX7 isoforms. (**A**) Representative confocal microscopic images of HEK-293 cells transfected with three different plasmids, mock or encoding hCBX7v1 or hCBX7v3. At Day 3, cells were stained with an anti-CBX7 antibody and phalloidin. DAPI (blue). (**B**) Representative confocal microscopic images of MEFs transduced with Ad-Mock, Ad-mCBX7v1, and Ad-mCBX7, respectively. At Day 3, cells were stained with an anti-CBX7 antibody and phalloidin. DAPI (blue).

To gain insights into how CBX7 isoforms regulate cell proliferation, we examined their subcellular localization via immunocytochemistry (ICC). For detection of CBX7 isoform proteins, we used an anti-CBX7 antibody. Phalloidin was used to visualize the cytoplasm. For the human cell study, we transfected HEK-293 cells with plasmid vectors inducing overexpression of hCBX7v1 and hCBX7v3. The plasmids also encode mCherry, a red fluorescent protein, downstream of an internal ribosomal entry site (IRES). Thus, cells transfected with plasmids express mCherry. Fluorescent microscopic imaging showed weak nuclear expression of CBX7 in cells transfected with a mock plasmid (Fig. 6A, upper panel). In the cells transfected with the hCBX7v1 plasmid, CBX7 proteins were localized to the nucleus (Fig. 6A, middle panel). In the cells transfected with the hCBX7v3 plasmid, CBX7 proteins were mainly localized to the cytoplasm while detected in nuclei in some of the cells (bottom panel). The nuclear proteins could be endogenous CBX7 which was also detected in cells transfected with the mock plasmid (Fig. 6A, upper panel).

For the mouse cell study, we infected MEFs with adenoviruses inducing overexpression of mCbx7 and mCbx7v1 and immunostained for CBX7. Fluorescent microscopic examination demonstrated weak nuclear and cytoplasmic expression of CBX7 in cells infected with the Ad-Mock virus (Fig. 6B, upper panel). CBX7 proteins produced by Ad-mCBX7v1 virus were localized to the nucleus (Fig. 6B, bottom panel). In the cells transfected with the Ad-mCBX7 virus, CBX7 proteins were mainly localized to the cytoplasm while some were detected in the nucleus (middle panel). The nuclear proteins could be endogenous CBX7 which was also detected in cells infected with Ad-Mock virus (Fig. 6B, upper panel). These results suggest that p22^CBX7^ is localized to the cytoplasm whereas 36^CBX7^ is localized to the nucleus.

## Discussion

This study for the first time demonstrated that CBX7 isoforms exhibit distinct characteristics in terms of expression patterns in response to different serum concentrations (Fig. 4), functions in proliferation (Fig. 5) and subcellular localization (Fig. 6). We newly identified mouse CBX7 transcript variant 1 (mCbx7v1), which is homologous to the human CBX7 gene (hCBX7v1) and is expressed in various mouse organs. We revealed that mCbx7v1 and hCBX7v1 encode a 36 kDa protein (p36^CBX7^) whereas mCbx7 and hCBX7v3 encode a 22 kDa protein (p22^CBX7^). This study further demonstrated that p36^CBX7^ was localized to the nucleus and endogenously expressed in proliferating cells whereas p22^CBX7^ was localized to the cytoplasm, induced by serum starvation in both human and mouse cells, and inhibited cell proliferation.

The roles of CBX7 have been extensively studied in both human and mice, but functional differences between its isoforms have not been described. The most notable finding of this study is that the original mCbx7 is not the functional homologue of hCBX7v1, but a newly discovered isoform of mouse CBX7, mCBX7v1, is (Figs. 1 and 3). A previous report showed that the mouse version of CBX7 is just smaller than the human one and their function is the same for cell proliferation, although the exact molecular weights were not specified^21^. However, we found that it is the mCbx7v1 gene that is homologous to the hCBX7v1 gene and is responsible for the conventionally known functions^21^. These two p36^CBX7^ homologues are endogenously expressed in proliferative cells such as MEFs and HEK-293 cells, suggesting their role in normal cell growth. This observation is consistent with the previous report that CBX7 is required for normal cell growth^21,24^.

On the other hand, the originally reported mCBX7 represses cell proliferation, and its human counterpart is hCBX7v3, whose function was first reported in this study. Our study showed that these two p22^CBX7^ were induced by serum starvation and inhibited proliferation of MEFs and HEK-293 cells. Serum starvation makes cells exit the cell cycle and enter G0 phase, also known as quiescence^40^. Thus, we speculate that p22^CBX7^ plays a crucial role in inducing or maintaining the non-proliferative state of cells. These findings are summarized in Table 1.

**Table 1:** Summary of characteristics of CBX7 isoforms in human and mouse.

Human isoform	Mouse isoform	Molecular weight	Subcellular localization	Expression pattern	Function
hCBX7v1	mCBX7v1	36 kDa	Nucleus	Endogenously expressed during normal growth	Required for normal cell growth
hCBX7v3	mCBX7	22 kDa	Cytoplasm	Induced by serum starvation	Inhibits cell proliferation

We speculate that two different isoforms work in a compensatory (or complementary) manner to regulate cellular homeostasis. In Fig. 3B, overexpression of the long isoform resulted in induction of the short isoform at Day 5. Thus, we suspect that the induction of the short isoform in response to excessive proliferative stimulation (overexpression of the long isoform) is required to prevent transformation and uncontrolled cell proliferation. Defining molecular interplay or negative feedback mechanisms between CBX7 isoforms will be an interesting topic to study in the future. Furthermore, determining whether CBX7 isoforms are essential for the observed phenotypes requires loss-of-function analyses but there is an inherent technical hurdle: it is extremely challenging to knockdown only the short isoform specifically since any siRNA targeting the short form will eventually affect the long isoform as well.

Interestingly, CBX7 isoforms exhibited distinct subcellular localization. We demonstrated that p22^CBX7^ and 36^CBX7^ are localized to the cytoplasm and nucleus, respectively. Previous reports showed that CBX7 binds to chromatin in the nucleus to represses senescence- and apoptosis-related genes such as Ink4a/Arf and Trail, respectively^22–24^. In addition, reports showed that EZH2, a polycomb group protein, was localized to the cytoplasm as well as the nucleus^41,42^. However, cytoplasmic localization of CBX7 has not been reported. We suspect that p22^CBX7^ interacts with certain cytoplasmic proteins that are critical for cell cycle progression, leading to inhibition of proliferation.

We for the first time compared CBX7 isoforms of p36 and p22 side by side in both human and mouse. CBX7 has been reported to function as an oncogene or a tumor suppressor gene depending on its context and the tissue^30^. However, their molecular basis has not been carefully explored. We claim that CBX7 isoforms play differential roles in regulating cell proliferation. Our notion is supported by literature describing distinct roles of protein isoforms in diverse contexts^43–47^. Further studies could help us understand which isoforms of CBX7 play major roles in different types of cancer. In addition, this study will help develop isoform-selective inhibitors of CBX7 for treating cancers.

## Materials and Methods

### Mice

Mice were used in accordance with animal protocols approved by the Emory University Institutional Animal Care and Use Committee (IACUC). CD-1 mice were purchased from Charles River Laboratories (Wilmington, MA). Neonatal (P0) mice were euthanized and the heart tissues were collected for RNA extraction. Adult (3-month-old) mice were euthanized and tissues including the brain, heart, liver, lung, kidney, thymus, spleen, and skeletal muscle were collected for extraction of RNA and protein.

### Molecular cloning of the mCBX7v1 gene

Total RNA was isolated from heart tissues of neonatal (P0) CD-1 mice using a guanidinium extraction method^48^ combined with an RNA extraction kit (Qiagen, Venlo, Netherlands). Extracted RNA was reversely transcribed using Taqman Reverse Transcription Reagents (Applied Biosystems, Beverly, MA, USA, Cat# 4304134) according to the manufacturer’s instructions. The synthesized cDNA was used for PCR with the following conditions: denaturation for 5 min at 95 °C, 30 cycles of 1 min at 95 °C, 1 min at 60 °C, and 2 min at 72 °C, followed by a 10 min final extension step at 72 °C. Primers were designed based on the cDNA sequences of mouse CBX7 (GenBank Accession NM144811.3), using Primer-Blast. Primer sequences are shown in Supplementary table 1. These primers were predicted to produce an 808 bp product. An unexpected DNA band at ~1,100 bp was isolated using a QIAquick Gel Extraction Kit (Qiagen), subcloned into the pGEM-T vector (Promega, Madison, WI, USA) by the TA cloning method, and sequenced by the dideoxy-mediated chain termination method.

### Quantitative Real-time PCR

Total RNAs were isolated from various organs of CD-1 adult (3-month-old) mice using a guanidinium extraction method^48^ combined with an RNeasy Mini Kit (Qiagen). Extracted RNA was reversely transcribed using Taqman Reverse Transcription Reagents (Applied Biosystems 4304134) according to the manufacturer’s instructions. The synthesized cDNA was subjected to qRT-PCR. Primer sequences used for this experiment are shown in Supplementary Table 1. Quantitative assessment of RNA levels was performed using the ABI PRISM 7500 Sequence Detection System (Applied Biosystems). Relative mRNA expression was normalized to *Gapdh* (See Supplementary Table 1).

### Generation of recombinant adenovirus particles

Cloned mouse CBX7 cDNAs were subcloned into an adenoviral shuttle plasmid, pDC316 (Microbix Biosystems, Mississauga, ON, Canada). Both adenoviral genomic and shuttle plasmids were transfected into HEK-293 cells using Lipofectamine 3000 (Thermo Fisher Scientific, Waltham, MA, USA). Recombinant adenoviral particles were extracted from cell lysates and the titer of adenoviral particles was determined via counting infected colonies using an antibody-mediated detection method (Clontech, Mountain View, CA, USA, Cat# 632250).

### Cell culture

HEK-293 cells and MEFs were purchased from ATCC. These cells were maintained in DMEM high glucose media supplemented with 10% FBS, 1% glutamine, and 1% non-essential amino acids (NEAA). Adenoviral particles were used at ~3 × 10^4^ IFU/ml. Plasmid DNAs for mock, hCBX7v1, and hCBX7v3 were purchased from GeneCopoeia (Rockville, MD, EX-NEG-M83, EX-Y2668-M83, EX-Y5634-M83). Transfection of plasmid DNA was performed according to the manufacturer’s instructions (Thermo Fisher Scientific, Cat# L3000015).

### Western blot

Adult (3-month-old) mouse tissues were freshly collected and homogenized in the RIPA buffer supplemented with protease-inhibitor cocktail (Sigma, P8340) and incubated at 4 °C overnight. The lysates were clarified by centrifugation. HEK-293 cells or MEFs were lysed with RIPA buffer supplemented with protease-inhibitor cocktail on ice for 1 h and the lysates were clarified by centrifugation. Equal amounts of lysates were subjected to SDS-PAGE, transferred onto a nitrocellulose membrane, and blocked for 1 h at room temperature in Tris-buffered saline with 0.05% Tween-20 (TBST) and 5% non-fat milk. The membrane was subsequently incubated with anti-CBX7 (Abcam, Cambridge, United Kingdom, Cat# ab21873, 1:3000) and anti-β-actin (Cell Signaling Technology, Danvers, MA, USA, Cat# 4967, 3:1000) at 4 °C overnight. After washing with TBST, blots were incubated with the appropriate secondary antibodies for 1 h at room temperature and developed using ECL detection reagent (Thermo Fisher Scientific).

### MTT assay

Cells were seeded on 96 well plates at 1 ×10^3^ cells per well. HEK-293 cells were transfected on a 6-well plate, transferred to the 96 well plate, and cultured in DMEM high glucose media supplemented with 10% FBS, 1% glutamine, 1% non-essential amino acids (NEAA). On the following day, media was changed to FBS-free media to prevent overgrowth of HEK-293 cells. The cells were then cultured for 72 hrs. MEFs were infected with adenoviral particles at the time of seeding and incubated for 72 hrs in DMEM high glucose media supplemented with 10% FBS, 1% glutamine, 1% NEAA 3-(4,5-dimethylthiazol-2-yl)-2,5-diphenyltetrazolium bromide (MTT) reagent was added to the cell culture medium at a final concentration of 0.5 mg/ml. The plate was incubated at 37 °C for 2 hrs in the darkness. After removal of culture medium, cells were lysed by DMSO and color was measured at 570 nm.

### Immunocytochemistry

Cells were fixed in 4% PFA at room temperature for 10 minutes. The samples were then permeabilized/blocked with PBS containing 0.1% Triton X-100 and 2.5% BSA at room temperature for 1 hour. Samples were then incubated with anti-CBX7 (Abcam, Cat# ab21873, 1:100) at 4 °C overnight. The slides were washed three times with PBS containing 0.1% Tween 20 and incubated with appropriate secondary antibodies or phalloidin (Thermo Fisher Scientific, Cat# A12381) at room temperature for 1–2 hours. DAPI was used for nuclear staining. The samples were visualized under a Zeiss LSM 880 confocal laser scanning microscope (Carl Zeiss, Oberkochen, Germany).

### Statistical analyses

Investigators were blinded to the assessment of the analyses of cell experiments. All data were presented as mean ± standard error of the mean (s.e.m). For the MTT assay, one-way ANOVA was performed followed by Tukey HSD Post Hoc test (N = 15–20 (each group)). Each experiment was repeated three times.

## Supplementary information

Supplementary Information.
