# Supplementary material for: Mammalian CBX7 isoforms p36 and p22 exhibit differential responses to serum, varying functions for proliferation, and distinct subcellular localization
Source: Sci Rep. 2020 May 15;10:8061. doi: 10.1038/s41598-020-64908-2 (PMC7228926; doi:10.1038/s41598-020-64908-2)
Supplement: Supplementary file 1 — Supplementary Information. [file 41598_2020_64908_MOESM1_ESM.pdf]

Supplementary material

Mammalian CBX7 isoforms p36 and p22 exhibit differential responses to serum, varying functions for proliferation, and distinct subcellular localization

Kyu-Won Cho, Mark Andrade, Yu Zhang, Young-sup Yoon

A

Species	Length (amino acids)	NCBI	Genbank Accession No.	Corresponding sequence in ENSEMBL	Sequence identity between NCBI and ENSEMBL
human	251	CBX7 transcript variant 1	NM 175709.4	201	100.0%
	158	CBX7 transcript variant 3	NM 001346744.1	202	100.0%
mouse	251	CBX7 transcript variant 1	MN 581682	201	98.4%
	158	CBX7	NM 144811.3	202, 203	100.0%

B

```
mCbx7-201      MELSAIGEQVFAVESIRKKRVRKGKVEYLVKWKGWPPKYSTWEPEEHILDPRLVMAYEEK
mCbx7v1        MELSAIGEQVFAVESIRKKRVRKGKVEYLVKWKGWPPKYSTWEPEEHILDPRLVTAYKEK
               ****************************************************** **:**

mCbx7-201      EERDRASGYRKRGPKPRRLLLQRLYSMDLRSSHKAKGNEKLCFSLARPLRSGSPMGVVKA
mCbx7v1        EERDRASGYRKRGPKPRRLLLQRLYSMDLRSSHKAKGNEKLCFSLARPLGSGSPMGVVKA
               ************************************************* **********

mCbx7-201      GVAELVEKGPLVPTLPFPLRKARKAHKYLRLSRKKFPPRGPHLESHSHRRELSLQESAAP
mCbx7v1        GVAELVEKGPLVPTLPFPLRKARKAHKYLRLSHKKFPPRGPHLESHSHRRELSLQESAAP
               ********************************:***************************

mCbx7-201      DVVQTPGDWEPMEQAPEEEAEADLTNGPPPWTPTLPSSEVTVTDITANSVTVTFREAQAA
mCbx7v1        DVVQTPGDWEPMEQAPEEEAEADLTNGPPPWTPTLPSSEVTVTDITANSVTVTFREAQAA
               ************************************************************

mCbx7-201      EGFFRDRNEKL
mCbx7v1        EGFFRDRNEKL
               ***********
```

C

```
hCBX7-201      MELSAIGEQVFAVESIRKKRVRKGKVEYLVKWKGWPPKYSTWEPEEHILDPRLVMAYEEK
hCBX7-202      MELSAIGEQVFAVESIRKKRVRKGKVEYLVKWKGWPPKYSTWEPEEHILDPRLVMAYEEK
mCbx7-201      MELSAIGEQVFAVESIRKKRVRKGKVEYLVKWKGWPPKYSTWEPEEHILDPRLVMAYEEK
mCbx7-202      MELSAIGEQVFAVESIRKKRVRKGKVEYLVKWKGWPPKYSTWEPEEHILDPRLVMAYEEK
mCbx7v1        MELSAIGEQVFAVESIRKKRVRKGKVEYLVKWKGWPPKYSTWEPEEHILDPRLVTAYKEK
               ****************************************************** **:**

hCBX7-201      EERDRASGYRKRGPKPKRLLLQRLYSMDLRSSHKAKGKEKLCFSLTCPLGSGSPEGVVKA
hCBX7-202      EERDRASGYRKRGPKPKRLLLQ--------------------------------------
mCbx7-201      EERDRASGYRKRGPKPRRLLLQRLYSMDLRSSHKAKGNEKLCFSLARPLRSGSPMGVVKA
mCbx7-202      EERDRASGYRKRGPKPRRLLLQ--------------------------------------
mCbx7v1        EERDRASGYRKRGPKPRRLLLQRLYSMDLRSSHKAKGNEKLCFSLARPLGSGSPMGVVKA
               ****************:*****

hCBX7-201      GAPELVDKGPLVPTLPFPLRKPRKAHKYLRLSRKKFPPRGPNLESHSHRRELFLQEPPAP
hCBX7-202      -------------------------------------------------------EPPAP
mCbx7-201      GVAELVEKGPLVPTLPFPLRKARKAHKYLRLSRKKFPPRGPHLESHSHRRELSLQESAAP
mCbx7-202      -------------------------------------------------------ESAAP
mCbx7v1        GVAELVEKGPLVPTLPFPLRKARKAHKYLRLSHKKFPPRGPHLESHSHRRELSLQESAAP
                                                                      *  **

hCBX7-201      DVLQAAGEWEPAAQPPEEEADADLAEGPPPWTPALPSSEVTVTDITANSITVTFREAQAA
hCBX7-202      DVLQAAGEWEPAAQPPEEEADADLAEGPPPWTPALPSSEVTVTDITANSITVTFREAQAA
mCbx7-201      DVVQTPGDWEPMEQAPEEEAEADLTNGPPPWTPTLPSSEVTVTDITANSVTVTFREAQAA
mCbx7-202      DVVQTPGDWEPMEQAPEEEAEADLTNGPPPWTPTLPSSEVTVTDITANSVTVTFREAQAA
mCbx7v1        DVVQTPGDWEPMEQAPEEEAEADLTNGPPPWTPTLPSSEVTVTDITANSVTVTFREAQAA
               **:*: *:***  * *****:***::*******:***************:**********

hCBX7-201      EGFFRDRSGKF
hCBX7-202      EGFFRDRSGKF
mCbx7-201      EGFFRDRNEKL
mCbx7-202      EGFFRDRNEKL
mCbx7v1        EGFFRDRNEKL
               *******. *:
```

Supplementary Figure 1. Protein sequence comparison of CBX7 isoforms. A. Comparison of CBX7 isoform sequences available at NCBI and Ensembl. B. Protein sequence alignment of mCbx7-201 and mCbx7v1. C. Protein sequence alignment of CBX7 isoforms available at Ensembl together with mCbx7v1.

A

```
HOMO SAPIENS                MELSAIGEQVFAVESIRKKRVRKGKVEYLVKWKGWPPKYSTWEPEEHILDPRLVMAYEEK
MACACA MULATTA              MELSAIGEQVFAVESIRKKRVRKGKVEYLVKWKGWPPKYSTWEPEEHILDPRLVMAYEEK
MUS MUSCULUS                MELSAIGEQVFAVESIRKKRVRKGKVEYLVKWKGWPPKYSTWEPEEHILDPRLVTAYKEK
RATTUS NORVEGICUS           MELSAIGEQVFAVESIRKKRVRKGKVEYLVKWKGWPPKYSTWEPEEHILDPRLVMAYEEK
CANIS LUPUS FAMILIARIS      MELSAIGEQVFAVESIRKKRVRKGKVEYLVKWKGWPPKYSTWEPEEHILDPRLVMAYEEK
BOS TAURUS                  MELSAIGEQVFAVESIRKKRVRKGKVEYLVKWKGWPPKYSTWEPEEHILDPRLVMAYEEK
OVIS ARIES                  MELSAIGEQVFAVESIRKKRVRKGKVEYLVKWKGWPPKYSTWEPEEHILDPRLVMAYEEK
SUS SCROFA                  MELSAIGEQVFAVESIRKKRVRKGKVEYLVKWKGWPPKYSTWEPEEHILDPRLVMAYEEK
EQUUS CABALLUS              MELSAIGEQVFAVESIRKKRVRKGKVEYLVKWKGWPPKYSTWEPEEHILDPRLVMAYEEK
                            ****************************************************** **:**

HOMO SAPIENS                EERDRASGYRKRGPKPKRLLLQRLYSMDLRSSHKAKGKEKLCFSLTCPLGSGSPEGVVKA
MACACA MULATTA              EERDRASGYRKRGPKPKRLLLQRLYSMDLRSSHKAKGKEKLCFSLTCPLGSGSPEGVVKA
MUS MUSCULUS                EERDRASGYRKRGPKPRRLLLQRLYSMDLRSSHKAKGNEKLCFSLARPLGSGSPMGVVKA
RATTUS NORVEGICUS           EEKDRASGYRKRGPKPKRLLLQRLYSMDLRSSHKAKGKEKLCFSLACPLGNGSPKGVVKA
CANIS LUPUS FAMILIARIS      EERDRASGYRKRGPKPKRLLLQRLYSMDLRSSHKAKGKEKLCFSLTRPLGSGSPEGVVKA
BOS TAURUS                  EERDRASGYRKRGPKPKRLLLQRLYSMDLRSSHKAKGKEKLCFSLTRPLGSGSPKGVVKA
OVIS ARIES                  EERDRASGYRKRGPKPKRLLLQRLYSMDLRSSHKAKGKEKLCFSLTRPLGSGSPEGVVKA
SUS SCROFA                  EERDRASGYRKRGPKPKRLLLQRLYSMDLRSSHKAKGKEKLCFSLARPLGSGSPEGVVKA
EQUUS CABALLUS              EERDRASGYRKRGPKPKRLLLQRLYSMDLRSSHKAKGKEKLCFSLTRPLGSGSPEGVVKA
                            **:*************:********************:*******: ***.*** *****

HOMO SAPIENS                GAPELVDKGPLVPTLPFPLRKPRKAHKYLRLSRKKFPPRGPNLESHSHRRELFLQEPPAP
MACACA MULATTA              GAPELVDKGPLVPTLPFPLRKPRKAHKYLRLSRKKFPPRGPNLESHSHRRELFLQEPPAP
MUS MUSCULUS                GVAELVEKGPLVPTLPFPLRKARKAHKYLRLSHKKFPPRGPHLESHSHRRELSLQESAAP
RATTUS NORVEGICUS           GAAELVEKGPLGPTLPFPLRKPHKAHKYLRLSHKKFPPCGSHLESHSHRRELSLQESAAP
CANIS LUPUS FAMILIARIS      GAP-MVDKGPMVPALPFPLRKPRKAHKYLRLSRKKFPPRGPDLESHSHPRELFLQEPAAP
BOS TAURUS                  GAPELADKGPLVPTLPFPLRKPRKAHKYLRLSRKKFPPRGPNLESHSHRRELFLQESPAQ
OVIS ARIES                  GAPELADKGPLVPTLPFPLRKPRKAHKYLRLSRKKFPPRGPNLESHSHRRELFLQESPAQ
SUS SCROFA                  GAPELADKGPLVPTLPFPLRKPRKAHKYLRLSRKKFPPRGPNLESHSHRRELFLQESPAP
EQUUS CABALLUS              GAPELVDKGPLVPALPFSLRKPRKAHKYLRLSRKKFPPRGPNLESHSHRRELFLQESPAP
                            *.  :.:***: *:*** *** :*********:***** * .****** *** ***  *

HOMO SAPIENS                DVLQAAGEWEPAAQPPEEEADADLAEGPPPWTPALPSSEVTVTDITANSITVTFREAQAAEGFFRDRSGKF
MACACA MULATTA              DVLQAAGEWEPAAQPPEEEADADLAEGPPPWTPALPSSEVTVTDITANSITVTFREAQAAEGFFRDRSGKF
MUS MUSCULUS                DVVQTPGDWEPMEQAPEEEAEADLTNGPPPWTPTLPSSEVTVTDITANSVTVTFREAQAAEGFFRDRNEKL
RATTUS NORVEGICUS           DVLQATGDWEPVEQPPEEEAEADLTNGPPPWTPMLPSSEVTVTDITANSVTVTFREAQAAEGFFRDRSGKL
CANIS LUPUS FAMILIARIS      DVLQAASEWEPAEQPPEE-EDADLAEGPPPWTPVLPASEVTVTDITANSVTVTFREAQAAEGFFRDRGGKF
BOS TAURUS                  DVLQAASEWEPAEQPPEEEAEADLGEGPPSWTPTLPSSEVTVTDITANSITVTFREAQAAEGFFRDRSGKF
OVIS ARIES                  DVLQAASEWEPAEQPPEEEAEADLGEGPPSWTPTLPSSEVTVTDITANSITVTFREAQAAEGFFRDRSGKF
SUS SCROFA                  DVLQAASEWEPAEQPPEEEADADLAEGPPSWTPTLPSSEVTVTDITANSITVTFREAQAAEGFFRDRGGKF
EQUUS CABALLUS              DVLQATGEWEPAEQPPEEEADADLAEGPPPWTPTLPPSEVTVTDITANSITVTFREAQAAEGFFRDRSGKF
                            **:*: .:***  * ***  :*** :*** *** ** ************:*****************  *:
```

B

```
HOMO SAPIENS                MELSAIGEQVFAVESIRKKRVRKGKVEYLVKWKGWPPKYSTWEPEEHILDPRLVMAYEEK
MACACA MULATTA              MELSAIGEQVFAVESIRKKRVRKGKVEYLVKWKGWPPKYSTWEPEEHILDPRLVMAYEEK
MUS MUSCULUS                MELSAIGEQVFAVESIRKKRVRKGKVEYLVKWKGWPPKYSTWEPEEHILDPRLVMAYEEK
RATTUS NORVEGICUS           MELSAIGEQVFAVESIRKKRVRKGKVEYLVKWKGWPPKYSTWEPEEHILDPRLVMAYEEK
CANIS LUPUS FAMILIARIS      MELSAIGEQVFAVESIRKKRVRKGKVEYLVKWKGWPPKYSTWEPEEHILDPRLVMAYEEK
BOS TAURUS                  MELSAIGEQVFAVESIRKKRVRKGKVEYLVKWKGWPPKYSTWEPEEHILDPRLVMAYEEK
OVIS ARIES                  MELSAIGEQVFAVESIRKKRVRKGKVEYLVKWKGWPPKYSTWEPEEHILDPRLVMAYEEK
SUS SCROFA                  MELSAIGEQVFAVESIRKKRVRKGKVEYLVKWKGWPPKYSTWEPEEHILDPRLVMAYEEK
EQUUS CABALLUS              MELSAIGEQVFAVESIRKKRVRKGKVEYLVKWKGWPPKYSTWEPEEHILDPRLVMAYEEK
                            ************************************************************

HOMO SAPIENS                EERDRASGYRKRGPKPKRLLLQEPPAPDVLQAAGEWEPAAQPPEEEADADLAEGPPPWTP
MACACA MULATTA              EERDRASGYRKRGPKPKRLLLQEPPAPDVLQAAGEWEPAAQPPEEEADADLAEGPPPWTP
MUS MUSCULUS                EERDRASGYRKRGPKPRRLLLQESAAPDVVQTPGDWEPMEQAPEEEAEADLTNGPPPWTP
RATTUS NORVEGICUS           EEKDRASGYRKRGPKPKRLLLQESAAPDVLQATGDWEPVEQPPEEEAEADLTNGPPPWTP
CANIS LUPUS FAMILIARIS      EERDRASGYRKRGPKPKRLLLQEPAAPDVLQAASEWEPAEQPPEEE-DADLAEGPPPWTP
BOS TAURUS                  EERDRASGYRKRGPKPKRLLLQESPAQDVLQAASEWEPAEQPPEEEAEADLGEGPPSWTP
OVIS ARIES                  EERDRASGYRKRGPKPKRLLLQESPAQDVLQAASEWEPAEQPPEEEAEADLGEGPPSWTP
SUS SCROFA                  EERDRASGYRKRGPKPKRLLLQESPAPDVLQAASEWEPAEQPPEEEADADLAEGPPSWTP
EQUUS CABALLUS              EERDRASGYRKRGPKPKRLLLQESPAPDVLQATGEWEPAEQPPEEEADADLAEGPPPWTP
                            **:*************:******  * **:*: .:***  * **** :*** :*** ***

HOMO SAPIENS                ALPSSEVTVTDITANSITVTFREAQAAEGFFRDRSGKF
MACACA MULATTA              ALPSSEVTVTDITANSITVTFREAQAAEGFFRDRSGKF
MUS MUSCULUS                TLPSSEVTVTDITANSVTVTFREAQAAEGFFRDRNEKL
RATTUS NORVEGICUS           MLPSSEVTVTDITANSVTVTFREAQAAEGFFRDRSGKL
CANIS LUPUS FAMILIARIS      VLPASEVTVTDITANSVTVTFREAQAAEGFFRDRGGKF
BOS TAURUS                  TLPSSEVTVTDITANSITVTFREAQAAEGFFRDRSGKF
OVIS ARIES                  TLPSSEVTVTDITANSITVTFREAQAAEGFFRDRSGKF
SUS SCROFA                  TLPSSEVTVTDITANSITVTFREAQAAEGFFRDRGGKF
EQUUS CABALLUS              TLPPSEVTVTDITANSITVTFREAQAAEGFFRDRSGKF
                             ** ************:*****************  *:
```

Supplementary Figure 2. Protein sequence alignment of CBX7 isoforms among different mammals. Alignment of CBX7 for 251 a.a. (A) and for 158 a.a. (B) isoforms.

hCBX7v1

Predicted NLSs in query sequence

```
MELSAIGEQVFAVESIRKKRVRKGKVEYLVKWKGWPPKYSTWEPEEHILD  50
PRLVMAYEEKEERDRASGYRKRGPKPKRLLLQRLYSMDLRSSHKAKGKEK 100
LCFSLTCPLGSGSPEGVVKAGAPELVDKGPLVPTLPFPLRKPRKAHKYLR 150
LSRKKFPPRGPNLESHSHRRELFLQEPPAPDVLQAAGEWEPAAQPPEEEA 200
DADLAEGPPPWTPALPSSEVTVTDITANSITVTFREAQAAEGFFRDRSGK 250
F                                                  251
```

Predicted bipartite NLS		
Pos.	Sequence	Score
63	RDRASGYRKRGPKPKRLLLQRLYSMDLRSSHKAKGK	6
70	RKRGPKPKRLLLQRLYSMDLRSSHKAKGKEKLCFS	5
115	EGVVKAGAPELVDKGPLVPTLPFPLRKPRKA	6.2

mCBX7v1

Predicted NLSs in query sequence

```
MELSAIGEQVFAVESIRKKRVRKGKVEYLVKWKGWPPKYSTWEPEEHILD  50
PRLVTAYKEKEERDRASGYRKRGPKPRRLLLQRLYSMDLRSSHKAKGNEK 100
LCFSLARPLGSGSPMGVVKAGVAELVEKGPLVPTLPFPLRKARKAHKYLR 150
LSHKKFPPRGPHLESHSHRRELSLQESAAPDVVQTPGDWEPMEQAPEEEA 200
EADLTNGPPPWTPTLPSSEVTVTDITANSVTVTFREAQAAEGFFRDRNEK 250
L                                                  251
```

Predicted bipartite NLS		
Pos.	Sequence	Score
63	RDRASGYRKRGPKPRRLLLQRLYSMDLRSSHKAKG	5.9
116	GVVKAGVAELVEKGPLVPTLPFPLRKARKA	6.8

hCBX7v3

Predicted NLSs in query sequence

```
MELSAIGEQVFAVESIRKKRVRKGKVEYLVKWKGWPPKYSTWEPEEHILD  50
PRLVMAYEEKEERDRASGYRKRGPKPKRLLLQEPPAPDVLQAAGEWEPAA 100
QPPEEEADADLAEGPPPWTPALPSSEVTVTDITANSITVTFREAQAAEGF 150
FRDRSGKF                                           158
```

Predicted bipartite NLS		
Pos.	Sequence	Score

mCBX7

Predicted NLSs in query sequence

```
MELSAIGEQVFAVESIRKKRVRKGKVEYLVKWKGWPPKYSTWEPEEHILD  50
PRLVMAYEEKEERDRASGYRKRGPKPRRLLLQESAAPDVVQTPGDWEPME 100
QAPEEEAEADLTNGPPPWTPTLPSSEVTVTDITANSVTVTFREAQAAEGF 150
FRDRNEKL                                           158
```

Predicted bipartite NLS		
Pos.	Sequence	Score

Score meaning

- 8-10: exclusively localized to the nucleus
- 6-7: partially localized to the nucleus
- 3-5: localized to both the nucleus and the cytoplasm
- 1-2: localized to the cytoplasm

Supplementary Figure 3. Prediction of nuclear localization signals (NLSs) in CBX7 isoforms via cNLS Mapper.

A

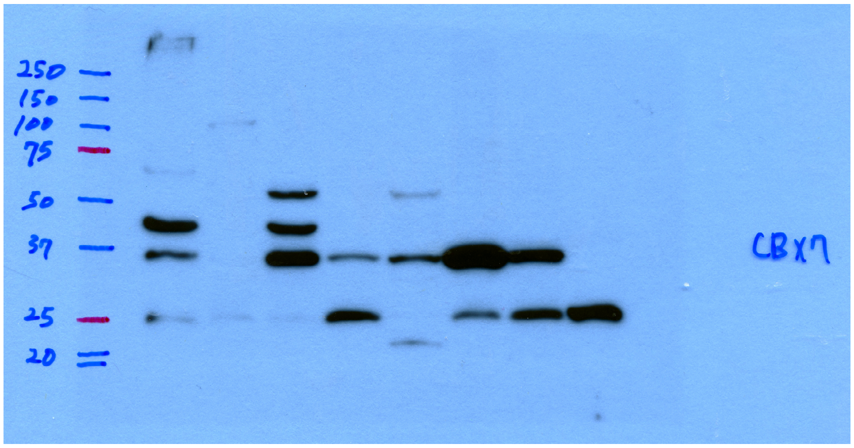

B

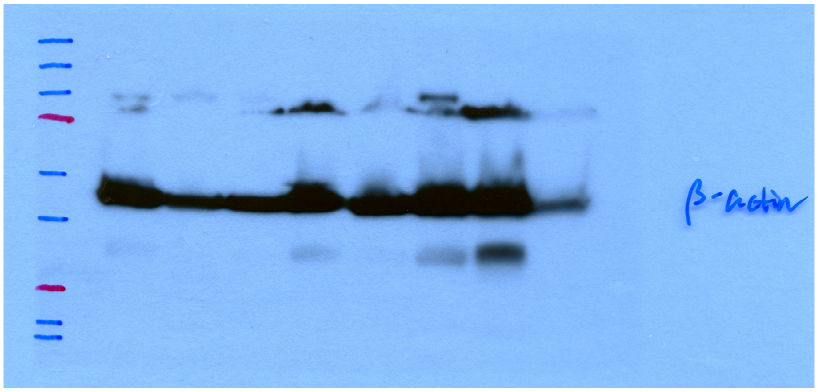

Supplementary Figure 4. Uncropped full-length blots for Figure 2C. Each blot represents a single gel. A. CBX7. B. β-actin.

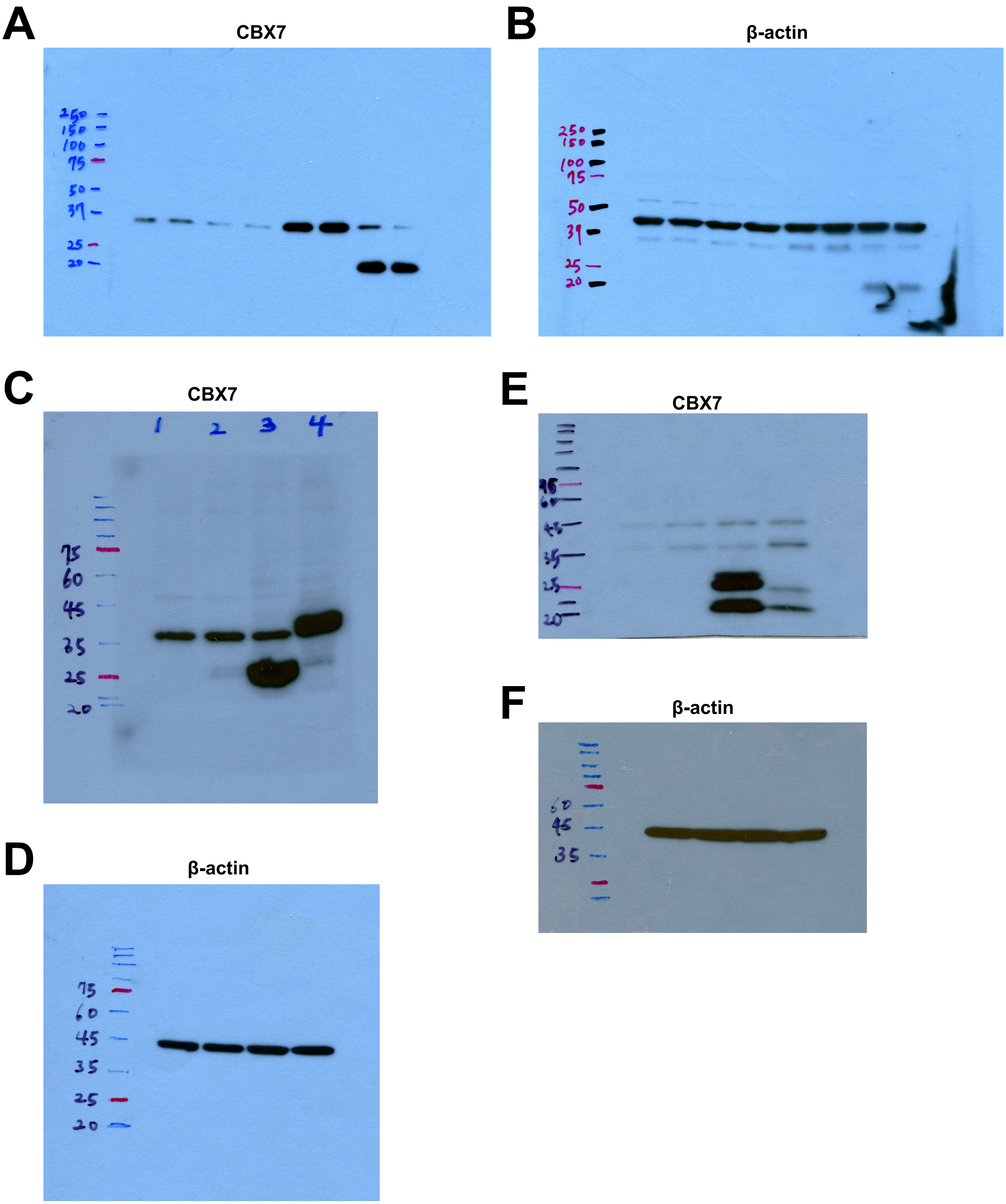

Supplementary Figure 5. Uncropped full-length blots for Figure 3. A-B. The original blot for Figure 3A. C-D. The original blot for Figure 3B (left). E-F. The original blot for Figure 3B (right). Each blot represents a single gel.

A

CBX7

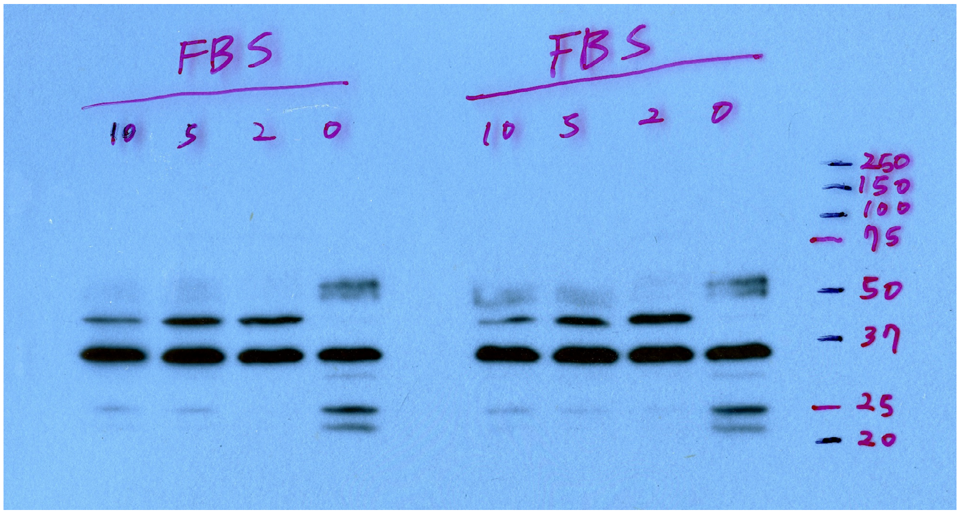

B

β-actin

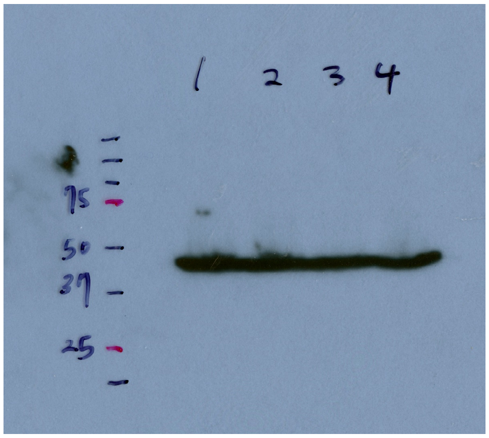

C

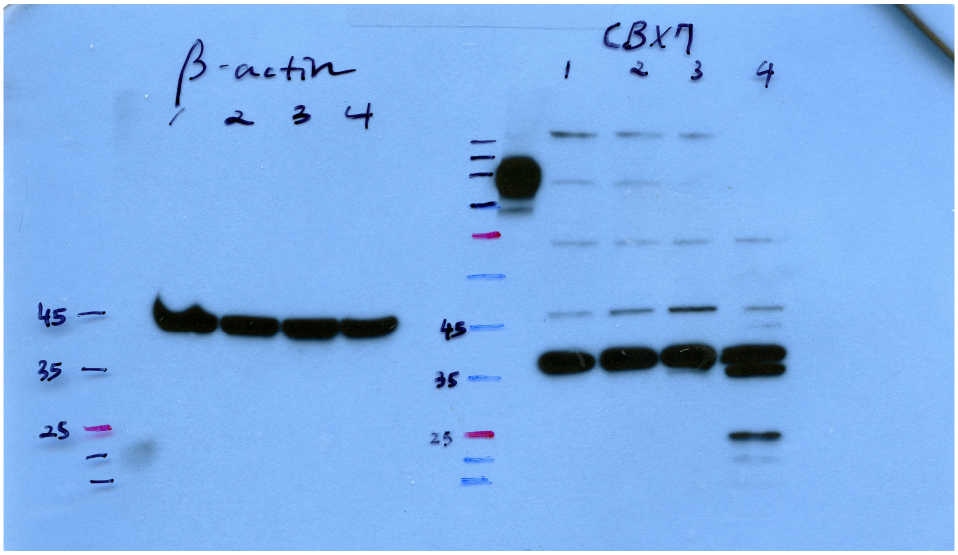

Supplementary Figure 6. Uncropped full-length blots for Figure 4. A-B. The original blot for Figure 4A. The original blot for Figure 4B. Each blot represents a single gel.

Supplementary Table 1. Sequence of primers used in this study.

Purpose	Forward (5' → 3')	Reverse (5' → 3')
Cloning of mCbx7v1	ATGGAGCTGTCAGCCATAGG	TGTCCGGATGTGTTCATGGG
qPCR for mCbx7	TGTGCTACAGGAGTCAGCAG	GAGTTGGCGGTGATGTCAGT
qPCR for mCbx7v1	CAAAGCCAAGGGCAATGAGAA	GGCGGAAACTTCTTGTGTGA
qPCR for Gapdh	ATGACCACAGTCCATGCCATC	CCTGCTTCACCACCTTCTTG
